# Plasmacytoid Variant Urothelial Cell Carcinoma: A Case of a Histological Variant of Urinary Bladder Cancer With Aggressive Behavior

**DOI:** 10.7759/cureus.36278

**Published:** 2023-03-17

**Authors:** Mosaad I Alshahwan, Musaad M Bin Dukhi, Sultan N Alotaibi, Rakan Aldarrab, Naif A Alhefdhi, Nourah Al Oudah, Saad Abumelha

**Affiliations:** 1 College of Medicine, King Saud Bin Abdulaziz University for Health Sciences, Riyadh, SAU; 2 Medicine, King Saud Bin Abdulaziz University for Health Sciences, Riyadh, SAU; 3 Urology, King Abdulaziz Medical City, Riyadh, SAU; 4 Urology, Security Forces Hospital, Riyadh, SAU; 5 Pathology and Laboratory Medicine, King Abdulaziz Medical City, Riyadh, SAU

**Keywords:** transitional cell carcinoma, diagnosis, plasmacytoid tumors, bladder cancer, plasmacytoid variant urothelial carcinoma

## Abstract

This paper presents a case report of plasmacytoid variant urothelial carcinoma (PVUC), a rare form of transitional cell carcinoma. PVUC is known for its unique clinical features, aggressive behavior, and poor survival rates. PVUC comprises less than 3% of all bladder tumors, and its diagnosis is often difficult due to its resemblance to other forms of bladder cancer. It requires a staging workup to rule out metastasis, relies heavily on immunostaining and histopathological analysis for diagnosis, and requires a multidisciplinary approach with early aggressive treatment, including cisplatin-based chemotherapy following surgery. This report highlights the importance of understanding rare variants of bladder cancer to ensure timely and accurate diagnosis and appropriate treatment planning. We report here a case of a 75-year-old male with multiple comorbidities who presented with hematuria and was diagnosed with urothelial carcinoma plasmacytoid type, which was initially treated with transurethral resection but later found to be unresectable and treated with palliative chemotherapy and radiation therapy. Eventually, the patient passed away three years after the diagnosis.

## Introduction

Bladder cancer is one of the most common cancers of the genitourinary tract [1]. Bladder cancer accounts for 549,000 new cases and about 200,000 deaths per year, which made it one of the most common cancers worldwide [2]. Bladder cancer has several subtypes, with urothelial carcinoma being the most common subtype, whereas squamous cell carcinoma, small cell carcinoma, adenocarcinoma, and sarcoma are infrequent [1]. Tobacco smoking is the main risk factor that is linked to the occurrence of bladder cancer, particularly the urothelial carcinoma subtype [1]. Another risk factor contributing to the development of bladder cancer is gender. Males are three times more probable to develop bladder cancer than females [3-6]. Even though there isn’t much data on genetic inheritance related to bladder cancer, there is an increased risk of early-onset bladder cancer for people who have a positive family history [6-8]. The percentage of urothelial carcinoma is 90% of all bladder carcinoma, and it can be either muscle-invasive or non-muscle-invasive carcinoma [3]. A rare histological form of transitional cell carcinoma is the plasmacytoid variant of urothelial carcinoma (PUC) of the urinary bladder [9]. The data on PUC indicates that this type of neoplasia has a unique clinical feature. Furthermore, PUC showed aggressive behavior and a poor survival rate [10,11]. In 1991, Sahin et al. described the first case of PUC of the bladder in a 63-year-old man who was diagnosed with a bladder tumor and several lytic bone lesions with a histologic appearance that resembled multiple myeloma [12]. The stages also vary from diffuse to extensive infiltration of the bladder which presents like a “linitis plastica” penetration and solidifies and thickens the bladder wall [13]. Moreover, PUC has a worse prognosis than typical urothelial carcinoma due to a higher probability of advanced disease, surgical margin positivity, and metastasis upon presentation [13]. The PUC has a higher risk of involvement of the perivesical and ureteral margins with a roughly 10-fold elevation. In contrast, conventional urothelial carcinoma carries an 8.5-fold elevation to involve perivesical and ureteral margins [13]. Upon the initial manifestation of the PUCs, extension to the peritoneum is observed [14]. The distinctive clinical features of PUC, including clinical presentation, tumor morphology, and molecular features, as well as its immunohistochemistry, can be confused with other forms [14]. Thus, knowledge of these uncommon variants is crucial to avoid a late or incorrect diagnosis [13]. In our paper, we present a case of transitional cell carcinoma, the plasmacytoid variant.

## Case presentation

**Figure 1:**
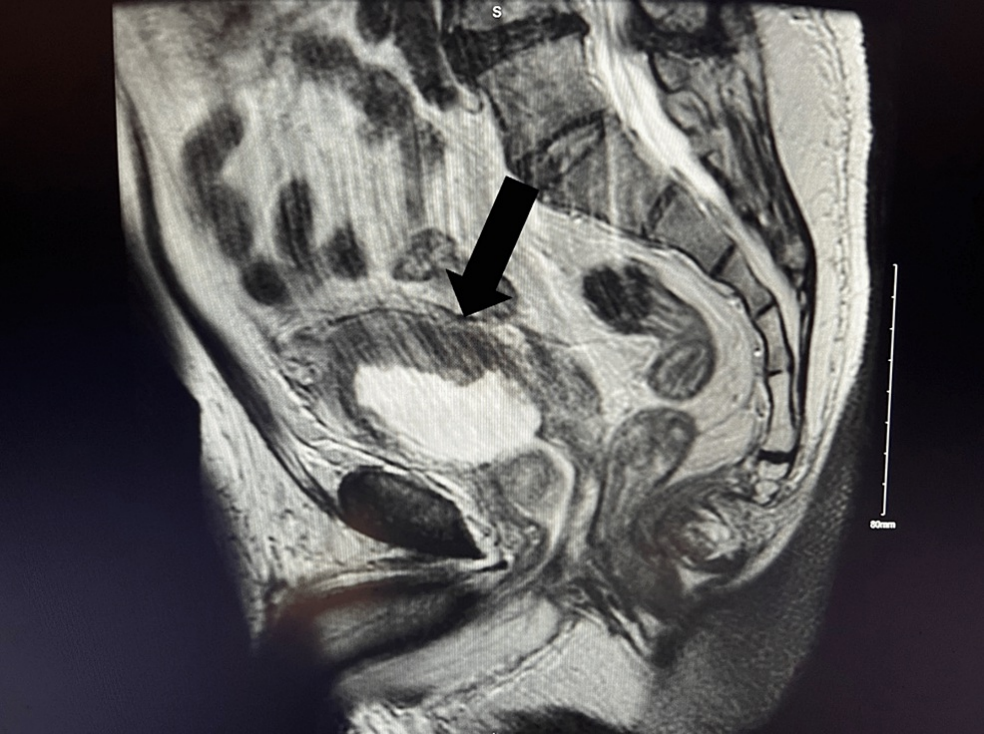
Sagittal view of the abdomen post transurethral resection of a bladder tumor (TURBT)

**Figure 2:**
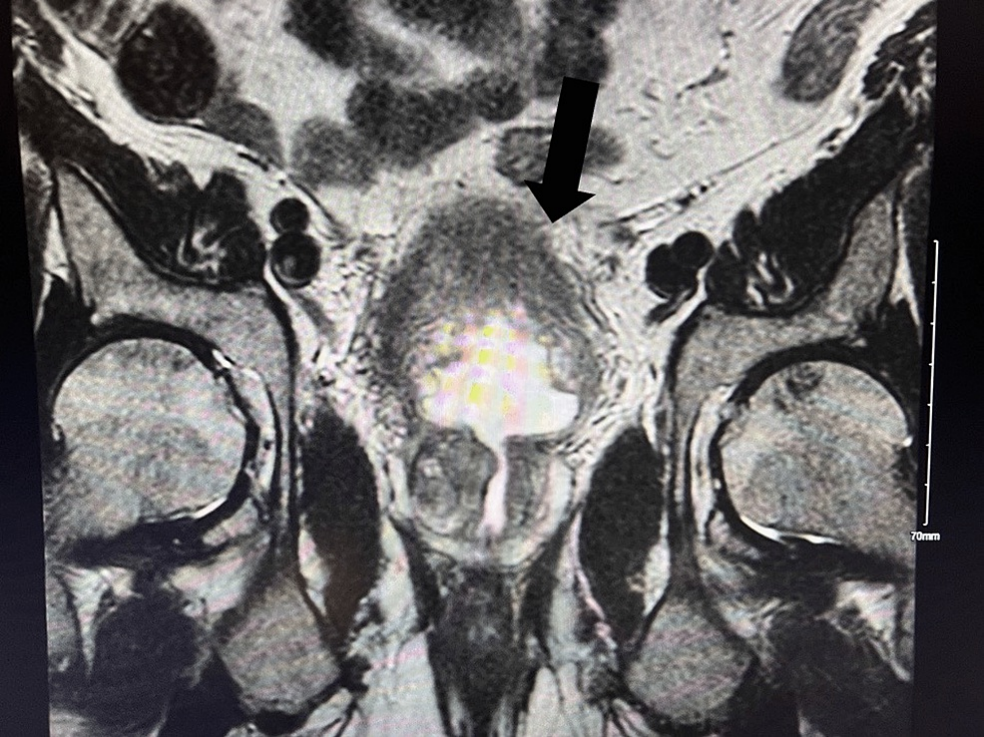
Coronal view of the abdomen post transurethral resection of a bladder tumor (TURBT)

 A 75-year-old male presented to the urology outpatient clinic on January 17, 2021, with dysuria, gross hematuria, frequency, and urgency. The patient had a weight loss of 4 kg over a one-month period. He was a chronic smoker for 50 years, and a known case of diabetes mellites, hypertension, dyslipidemia, benign prostatic hyperplasia, and chronic kidney disease (IIIb). The family history of the patient was reviewed, and it was unremarkable. On examination, the patient was alert, oriented, and vitally stable. A digital rectal exam (DRE) was painful to the patient, and it showed moderate enlargement of the prostate that extended to the lateral pelvic wall. His routine laboratory workup was done. An elevation in the creatinine level was noted. Cystoscopy was done on January 18, 2021, for the patient in the clinic under local anesthesia, and it showed normal urethra and prostate, suspicious lesions on the trigone, and the posterior bladder wall. A urine cytology study was done, and it showed squamous cells. Transurethral resection of the bladder tumor was done for him on February 4, 2021. Resection of the whole mass was done with deep cuts sent to histopathology; suspicious fecal matter was seen before starting the resection. A CT cystogram and MRI abdomen were done, which showed a thickening bladder wall and did not identify any fistulas (Figures 1-2).

**Figure 3:**
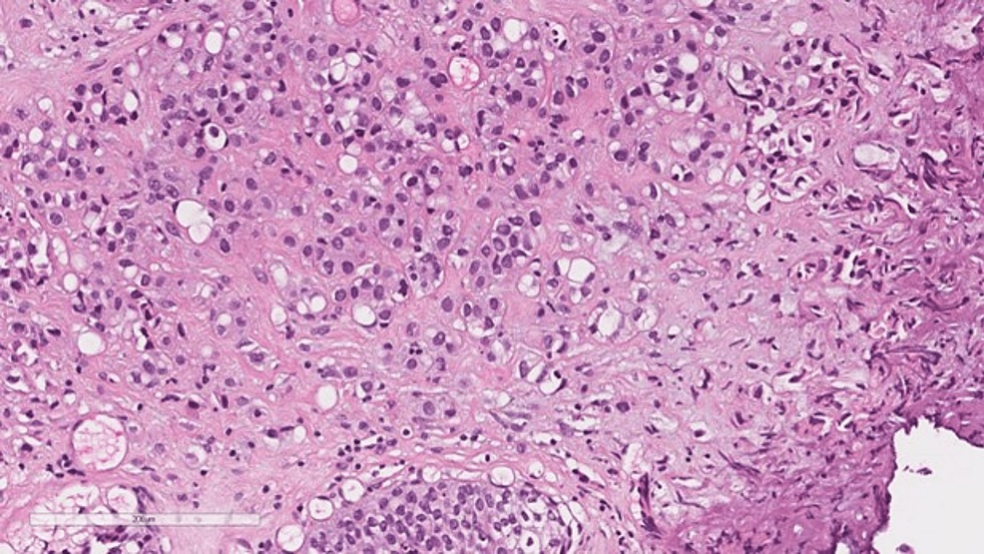
Higher magnification shows the infiltrating cells with eccentrically placed nuclei and abundant eosinophilic cytoplasm similar to plasmacytes, some with vacuoles

The histopathological examination showed urothelial carcinoma plasmacytoid type with a diffuse infiltrate (Figure 3).

**Figure 4:**
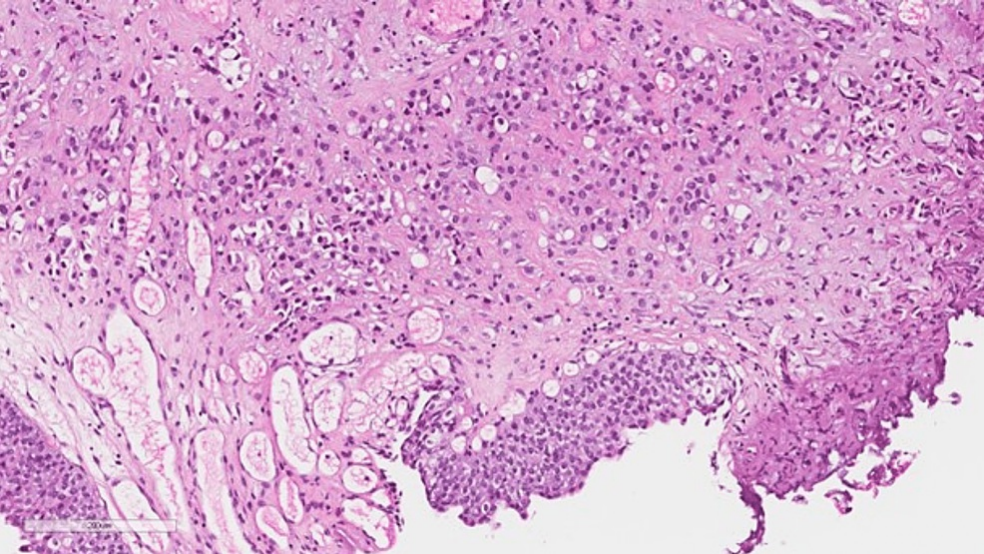
There is infiltrating single cell with plasmatic-looking cells in the lamina propria

The tumor invades through the lamina propria and muscularis propria while no lymph vascular invasion was identified (Figure 4).

**Figure 5:**
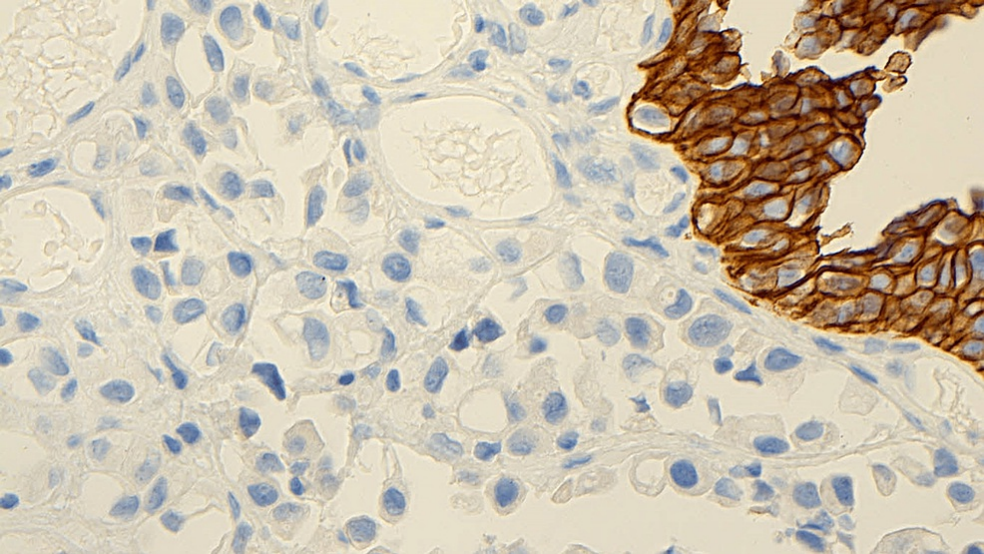
Tumor cells negative for E-cadherin while the lining urothelium is positive

The immunohistochemical profiles of the tumor cells manifested high ki67 >50%. The tumor showed positivity for CD138, HMW CK, CK20, and GATA 3. However, PSA, CDX2, P63, and E-cadherin showed negative results (Figure 5).

Staging was done for him, which came back negative for both bone and intra-abdominal metastasis. The case was discussed in our tumor board meeting and the decision was made to go for surgical resection. Laparotomy was done on July 2022. Upon exploration, the tumor was found to be unresectable due to its invasion into the anterior abdominal wall and the lateral pelvic wall so the patient was closed without any intervention. Postop, the patient was referred to oncology for palliative chemotherapy where he received six cycles of palliative gemcitabine and Carboplatin with a 25% dose reduction secondary to grade 4 myelotoxicity. The patient has been receiving avelumab after the stabilization of his condition. Then, the patient was referred to radiation oncology due to pain, for which he was given 21-Gy radiation therapy over three fractions. During follow-up, he developed bilateral hydronephrosis. Therefore, a bilateral nephrostomy was inserted, which result in the stabilization of the creatinine level. The final tumor node metastasis (TNM) staging for him was T4 N0 M0. The patient kept regular follow-ups with medical oncology till he passed away three years after the diagnosis. He was admitted due to small bowel obstruction for which he underwent exploratory laparotomy, right hemicolectomy, and the Hartmann procedure. The patient was kept in the ICU till his death, which was due to a cardiac arrest.

## Discussion

One percent to 3% of all bladder tumors are plasmacytoid [15]. Survival data indicated a tendency toward a shorter overall survival of individuals with PUC compared to those with conventional invasive urothelial carcinoma [10]. About 65% of the patients have hematuria when they presented initially [16]. Our patient was a 75-year-old male who presented with gross hematuria. Imaging shows a widespread thickening of the urinary bladder's wall. A noncontrast CT scan of our patient showed circumferential urinary bladder wall thickening. Histologically, plasmacytoid tumors are distinguished by small (2-3 times the size of a lymphocyte) nuclei that are eccentrically positioned with abandoned eosinophilic cytoplasm; cells are organized loosely, or "discohesively," and form dispersed cords [17]. On histopathological examination of our patient sample, there was urothelial carcinoma, diffuse plasmacytoid type. These tumors show a diffuse infiltrate under the microscope, with individual discohesive cells. Loss of E-cadherin-mediated cell adhesion is an attributing factor in plasmacytoid urothelial carcinoma's lack of cohesiveness and infiltrative appearance [18]. The intracytoplasmic vacuoles of plasmacytoid urothelial carcinoma may give them the appearance of signet ring cells [19,20]. Muscularis propria invasion and lamina propria invasion were seen in one of two cases of plasmacytoid variant urothelial carcinoma in a previous study [21]. In our patient, the tumor invaded through the lamina propria and muscularis propria while no lymphovascular invasion was identified. Fritsche et al. [22] proposed the theory suggesting plasmacytoid differentiation in urothelial cancer may be linked to E-cadherin loss. According to a review of the literature, 13 different cases of plasmacytoid urothelial cancer were stained for E-cadherin. Each of these cases lacked expression of E-cadherin [23-25]. In our patient, there was a lack of expression of E-cadherin. The rare and aggressive plasmacytoid form of urothelial carcinoma has plasmacytoid differentiation, and the cells are CK7, GATA3, CD138, and CK20 positive [26]. When compared to other urothelial malignancies, truncating CDH1 mutations were the sole distinctive mutations found in the plasmacytoid variant, making them the distinctive molecular characteristic of the variant. Additionally, these tumors express CD138, which is a diagnostic red flag [27]. The tumor cells in our patient had immunohistochemical profiles that showed an increase in proliferative marker Ki67>50. Additionally, it was noted that CD138, HMW CK, CK20, and GATA 3 were positive. PSA, CDX2, P63, and E-cadherin on the other hand, gave negative results. Because it is a rare and deadly tumor with a bad prognosis, a surgical procedure involving a multidisciplinary approach is warranted. Early radical cystectomy should be offered to the patients, which was planned for our patient but, unfortunately, the tumor was unresectable at the time of the surgery. Due to the lack of distinct margins of the specimen, resection is most often incomplete [28]. The use of transurethral resection or radical cystectomy with urinary diversion is an option for surgeons. In the indexed case, transurethral resection of a bladder tumor (TURBT) was done for the patient. Given the rarity of this histologic kind of tumor, there is no agreement on how it should be managed following surgical treatment. There are discussions of radiation therapy and chemotherapy [29]. The first line of treatment for medically fit patients is platinum-based combination chemotherapy, which includes carboplatin plus gemcitabine, or carboplatin, gemcitabine, and paclitaxel. Alternatively, a non-platinum-based combination (e.g., paclitaxel plus gemcitabine) or even monotherapy are different therapies for patients who cannot get cisplatin because of their medical fragility or comorbidities (e.g., gemcitabine) [30]. Following surgery, a platinum-based combination therapy regimen increases progression-free and overall survival [31]. According to some research, there is no survival difference between the plasmacytoid variant in patients treated with neoadjuvant chemotherapy and surgery compared to patients treated with surgery alone [32]. In the indexed case, palliative chemotherapy was given to him, where he received six cycles of palliative gemcitabine and carboplatin with a 25% dose reduction secondary to grade 4 myelotoxicity. Because of his pain, he was referred to radiation oncology, where he received 21-Gy radiation therapy over three fractions. A contrast-enhanced MRI of the abdomen and pelvis was performed as a follow-up procedure for our patient in the indexed case. It demonstrated a considerable improvement in the post-radiation, fibrosis-related diffuse thickening of the bladder wall.

## Conclusions

Urothelial carcinoma with the plasmacytoid variant is rare. In every situation, a staging workup is necessary to rule out metastasis. The diagnosis relies heavily on immunostaining and histopathological analysis. Loss of E-cadherin expression may also have a role in the aggressive nature of these forms of urothelial carcinoma, in addition to being a hallmark of plasmacytoid variant differentiation in urothelial carcinoma. A surgical procedure with a multidisciplinary approach is necessary because it is a rare and fatal tumor. It is a fast-growing disease and early aggressive treatment should be applied for the patients. Platinum-based combination therapy is the first line of treatment following surgery for patients who meet medical criteria.

## References

[1] Minoli M, Kiener M, Thalmann GN, Kruithof-de Julio M, Seiler R (2020). Evolution of urothelial bladder cancer in the context of molecular classifications. Int J Mol Sci.

[2] Bray F, Ferlay J, Soerjomataram I, Siegel RL, Torre LA, Jemal A (2018). Global cancer statistics 2018: GLOBOCAN estimates of incidence and mortality worldwide for 36 cancers in 185 countries. CA Cancer J Clin.

[3] Burger M, Catto JW, Dalbagni G (2013). Epidemiology and risk factors of urothelial bladder cancer. Eur Urol.

[4] Ferlay J, Colombet M, Soerjomataram I (2018). Cancer incidence and mortality patterns in Europe: estimates for 40 countries and 25 major cancers in 2018. Eur J Cancer.

[5] Berdik C (2017). Unlocking bladder cancer. Nature.

[6] Aben KK, Witjes JA, Schoenberg MP, Hulsbergen-van de Kaa C, Verbeek AL, Kiemeney LA (2002). Familial aggregation of urothelial cell carcinoma. Int J Cancer.

[7] Lin J, Spitz MR, Dinney CP, Etzel CJ, Grossman HB, Wu X (2006). Bladder cancer risk as modified by family history and smoking. Cancer.

[8] Murta-Nascimento C, Silverman DT, Kogevinas M (2007). Risk of bladder cancer associated with family history of cancer: do low-penetrance polymorphisms account for the increase in risk?. Cancer Epidemiol Biomarkers Prev.

[9] da Fonseca LG, Souza CE, Mattedi RL, Girardi DM, Sarkis ÁS, Hoff PM (2014). Plasmacytoid urothelial carcinoma: a case of histological variant of urinary bladder cancer with aggressive behavior. Autops Case Rep.

[10] Keck B, Stoehr R, Wach S (2011). The plasmacytoid carcinoma of the bladder—rare variant of aggressive urothelial carcinoma. Int J Cancer.

[11] Mai KT, Park PC, Yazdi HM (2006). Plasmacytoid urothelial carcinoma of the urinary bladder report of seven new cases. Eur Urol.

[12] Sahin AA, Myhre M, Ro JY, Sneige N, Dekmezian RH, Ayala AG (1991). Plasmacytoid transitional cell carcinoma. Report of a case with initial presentation mimicking multiple myeloma. Acta Cytol.

[13] Sood S, Paner GP (2019). Plasmacytoid urothelial carcinoma: an unusual variant that warrants aggressive management and critical distinction on transurethral resections. Arch Pathol Lab Med.

[14] Ricardo-Gonzalez RR, Nguyen M, Gokden N, Sangoi AR, Presti JC Jr, McKenney JK (2012). Plasmacytoid carcinoma of the bladder: a urothelial carcinoma variant with a predilection for intraperitoneal spread. J Urol.

[15] Fox MD, Xiao L, Zhang M (2017). Plasmacytoid urothelial carcinoma of the urinary bladder: a clinicopathologic and immunohistochemical analysis of 49 cases. Am J Clin Pathol.

[16] Yamamoto S, Ito T, Akiyama A (2001). Primary signet-ring cell carcinoma of the urinary bladder inducing renal failure. Int J Urol.

[17] Perrino CM, Eble J, Kao CS (2019). Plasmacytoid/diffuse urothelial carcinoma: a single-institution immunohistochemical and molecular study of 69 patients. Hum Pathol.

[18] Lim MG, Adsay NV, Grignon DJ, Osunkoya AO (2011). E-cadherin expression in plasmacytoid, signet ring cell and micropapillary variants of urothelial carcinoma: comparison with usual-type high-grade urothelial carcinoma. Mod Pathol.

[19] Lopez-Beltran A, Requena MJ, Montironi R, Blanca A, Cheng L (2009). Plasmacytoid urothelial carcinoma of the bladder. Hum Pathol.

[20] Ro JY, Shen SS, Lee HI (2008). Plasmacytoid transitional cell carcinoma of urinary bladder: a clinicopathologic study of 9 cases. Am J Surg Pathol.

[21] Yamaguchi K, Yoshihiro T, Ariyama H (2022). Potential therapeutic targets discovery by transcriptome analysis of an in vitro human gastric signet ring carcinoma model. Gastric Cancer.

[22] Fritsche HM, Burger M, Denzinger S, Legal W, Goebell PJ, Hartmann A (2008). Plasmacytoid urothelial carcinoma of the bladder: histological and clinical features of 5 cases. J Urol.

[23] Patriarca C, Di Pasquale M, Giunta P, Bergamaschi F (2008). CD138-positive plasmacytoid urothelial carcinoma of the bladder. Int J Surg Pathol.

[24] Sato K, Ueda Y, Kawamura K, Aihara K, Katsuda S (2009). Plasmacytoid urothelial carcinoma of the urinary bladder: a case report and immunohistochemical study. Pathol Res Pract.

[25] Keck B, Stöhr R, Goebell PJ, Fritsche HM, Wullich B, Hartmann A (2008). Plasmacytoid carcinoma. Five case reports of a rare variant of urothelial carcinoma [Article in German]. Pathologe.

[26] Thomas AA, Stephenson AJ, Campbell SC, Jones JS, Hansel DE (2009). Clinicopathologic features and utility of immunohistochemical markers in signet-ring cell adenocarcinoma of the bladder. Hum Pathol.

[27] Al-Ahmadie HA, Iyer G, Lee BH (2016). Frequent somatic CDH1 loss-of-function mutations in plasmacytoid variant bladder cancer. Nat Genet.

[28] Spinoit AF, Petit T, Elalouf V, Saint F, Petit J (2011). Signet-ring cell primitive bladder carcinoma: a rare and aggressive tumor [Article in French]. Prog Urol.

[29] Calabrò F, Sternberg CN (2009). Neoadjuvant and adjuvant chemotherapy in muscle-invasive bladder cancer. Eur Urol.

[30] Galsky MD, Hahn NM, Rosenberg J (2011). A consensus definition of patients with metastatic urothelial carcinoma who are unfit for cisplatin-based chemotherapy. Lancet Oncol.

[31] El Ammari JE, Ahsaini M, Riyach O (2013). Primary signet-ring cell carcinoma of the urinary bladder successfully managed with cisplatin and gemcitabine: a case report. J Med Case Rep.

[32] Li Q, Assel M, Benfante NE (2017). The impact of plasmacytoid variant histology on the survival of patients with urothelial carcinoma of bladder after radical cystectomy. Eur Urol Focus.

